# Sleep Delicious

**Published:** 1876-01

**Authors:** 


### SLEEP DELICIOUS.

WHAT person of mature years can look on a sleeping child, and not envy the unconscious luxury of that undisturbed repose, especially if it is one's own child. It is none other than a pure delight to the parental beholder.

A lady correspondent writes: "From utter exhaustion, I slept all night like an infant. How ineffably soothing and refreshing was that sleep, three nights since. This power of resting, even for one brief night, encouraged me greatly. I feel even now, wasted as I am, if I could only have refreshing sleep—if I could rest—I could get well."

The excellent writer was suffering from no specially dangerous or critical malady; but from a general derangement of the whole nervous system. The incident is recorded for the purpose of bringing to the reader's mind the duty of habitual thankfulness for any ability he may have to go to bed, to fall asleep within ten minutes, and know nothing more until the gray morning breaks—a deep and warm gratitude should well up constantly from a loving heart to the Giver of all Good for the unfelt bliss of a whole night's sleep.

Some persons are put to sleep by having the soles of the feet rubbed gently with a soft, bare hand, when opiates made wild.

We know of no better plan for securing good sleep to persons not specially invalids, than to observe the following:

1. Take a very light supper, not later than six P. M.

2. Heat the bare feet before a fire, for the last fifteen minutes before bed-time.

3. Occupy a large room, with a window or door partly open, and the fire-place unclosed.

4. Go to bed at a regular hour.

5. Get up the moment of waking next morning, at whatever time that may be.

6. Do not on any account sleep a moment in the day time.

The result of these observances will be, in all cases where there is not serious disease of body or mind, that the person will, in a few days, go to sleep promptly, and wake the very moment that nature has had all the repose needed.

